# Intragenic β-synuclein rearrangements in malignancy

**DOI:** 10.3389/fonc.2023.1167143

**Published:** 2023-05-12

**Authors:** Peifang Xiao, Nan Chen, Tingting Shao, Xinni Bian, Jie Miao, Jiajia Zheng, Xingping Lang, Yiting Wang, Xiaojun Chen, Liqin Jin, Shaoyan Hu, Sheng Xiao

**Affiliations:** ^1^ Department of Hematology, Children’s Hospital of Soochow University, Suzhou, China; ^2^ Department of Molecular Genetics, Suzhou Sano Precision Medicine Ltd, Suzhou, China; ^3^ Department of Pathology, Brigham and Women’s Hospital, Harvard Medical School, Boston, MA, United States

**Keywords:** *SNCB*, *ETV6*, *GPRIN1*, T-ALL, synuclein

## Abstract

The synuclein family, consisting of α-, β-, and γ-synuclein, is primarily expressed in neurons. Mutations of α- and β-synuclein have been linked to Parkinson’s disease and dementia with Lewy bodies, respectively. Recent studies have shown that synucleins are upregulated in various tumors, including breast, ovarian, meningioma, and melanoma, and high synuclein expression is associated with poor prognosis and drug resistance. We report a novel rearrangement of β-synuclein in a pediatric T-cell acute lymphoblastic leukemia (T-ALL) case, where β-synuclein (*SNCB*) is fused in-frame with ETS variant transcription factor 6 (*ETV6*), a gene frequently rearranged in acute leukemia including acute myeloid leukemia (AML), B-cell acute lymphoblastic leukemia (B-ALL), and T-ALL. An additional case of β-synuclein rearrangement was identified in a squamous cell carcinoma of the lung through analysis of the public TCGA database. Both rearrangements involve the C-terminal of β-synuclein. Since β-synuclein shares extensive amino acid similarities with α-synuclein and α-synuclein binds to 14-3-3, an important regulator of apoptosis, the rearranged β-synuclein may contribute to tumorigenesis by deregulating apoptosis. In addition, overexpression of synucleins has been shown to increase cell proliferation, suggesting that the rearranged β-synuclein may also deregulate the cell cycle.

## 1 Introduction

Synuclein is a group of three small acidic proteins, α-synuclein (140 amino acids), β-synuclein (134 amino acids), and γ-synuclein (127 amino acids). These proteins contain an N-terminal lysine-rich amphipathic α-helix domain, a central hydrophobic non-amyloid β component (NAC) domain, and a C-terminal structureless acidic region. The N-terminal domain is positively charged and capable of lipid binding to cell membranes, while the NAC domain, which is an antiparallel β-sheet structure, is involved in fibril formation and aggregation. The structureless acidic region interacts with the N-terminal domain to prevent synuclein aggregation. The C-terminal region is also responsible for protein interaction. α-synuclein and β-synuclein were first purified from the human brain and are both enriched in presynaptic terminals of neurons. γ-synuclein was first identified as the breast cancer-specific gene 1 due to its overexpression in breast tumors and is expressed in the nervous system, the endocrine tissues, and the kidneys and urinary tract.

The synucleins are mainly known for their role in synucleinopathies, a group of neurodegenerative disorders. Mutations of *SNCA*, encoding α-synuclein, or increased copies of the wild-type *SNCA* lead to early-onset autosomal dominant Parkinson’s disease, characterized by the loss of dopaminergic neurons in the substantia nigra. Two mutations of *SNCB*, encoding β-synuclein, the P123H and the V70M, are associated with dementia with Lewy bodies, a disorder closely related to Parkinson’s disease. Lewy bodies are protein aggregates in neurons and are pathological hallmarks in synucleinopathies, with α-synuclein being a major component.

The role of synucleins in cancer is supported by their overexpression and links to both disease progression and therapeutic response. α-synuclein is overexpressed in various types of cancer, including meningioma, pancreatic cancer, glioblastoma, ovarian cancer, colorectal cancer, and melanoma (1, 2). The expression of α-synuclein is associated with disease progression, as it is only expressed in meningiomas of grades 2 and 3, but not grade 1 (3). In pancreatic cancer, α-synuclein expression is correlated with neurotropism, a hallmark of tumor progression (4). There is limited evidence of β-synuclein’s involvement in cancer, but it is expressed in certain types of brain tumors, such as ependymoma, astrocytoma, oligodendroglioma, and medulloblastoma (5). γ-synuclein is overexpressed in many tumors, including breast, ovary, colon, liver, and cervical cancer, and its expression is higher in more advanced cancers (6, 7). *In vitro* assays and animal models suggest that γ-synuclein plays a crucial role in promoting tumor invasion and metastasis. Moreover, recent studies indicate that tumors with γ-synuclein overexpression are more resistant to chemotherapy and radiotherapy (8, 9).

We present a novel discovery of a β-synuclein rearrangement in a pediatric T-ALL tumor, where *SNCB* is fused to the well-known leukemic gene *ETV6*. We also found another case of *SNCB* rearrangement in a case of squamous lung cancer by searching the TCGA database. These are the first reported cases of tumors with *SNCB* rearrangements.

## 2 Materials and methods

### 2.1 Immunohistochemical studies

Immunohistochemistry (IHC) was performed on 5-μm tissue sections using the following protocol: (1) the slides were baked at 60 °C for 1 hour, (2) deparaffinized and rehydrated with 100% xylene, 100% ethanol, 70% ethanol, and running water, (3) blocked in a solution of 10% normal serum and 1% bovine serum albumin (BSA) in Tris-buffered saline, (4) incubated with primary antibodies for 2 hours, (5) endogenous peroxidase was blocked with 0.3% hydrogen peroxide, and (6) slides were incubated with horseradish peroxidase-labeled polymer (DAKO) according to the manufacturer’s instructions. The tissue sections were developed using 3,3’-diaminobenzidine (DAKO) as the chromogen and counterstained with Mayer’s hematoxylin.

### 2.2 Cytogenetics

The bone marrow specimens were processed as follows: (1) cells were collected in sodium heparin and counted using a TC-20 Automated Cell Counter (Bio-Rad, Hercules, CA), (2) 5 × 10^6^ cells were cultured overnight and treated with ethidium bromide (5 µg/mL) for 1.5 hours and Colcemid (0.5 µg/mL) for 20 minutes, (3) cells were incubated in a hypotonic solution, fixed, and metaphase spreading slides were manually prepared, (4) slides were baked at 70°C for 1 hour, (5) chromosomes were Giemsa-Trypsin banded and analyzed. For fluorescence *in situ* hybridization (FISH), a two-colored split apart probe for *ETV6* was added to metaphase slides, co-denatured, and hybridized at 37°C overnight. Post-hybridization wash was performed in 0.4 × SSC/0.3% NP-40 at 73°C for 3 minutes, and slides were counterstained with DAPI.

### 2.3 Targeted RNA next-generation sequencing

Total RNA was isolated from the bone marrow aspirate using the TRIZOL reagent (ThermoFisher, Waltham, MA) according to the manufacturer’s instructions. The reverse transcription, end repairing, dA-tailing, and adaptor ligation were performed following standard NGS protocols (NEB, Cat E7771 and E6111, Ipswich, MA, USA). A group of 75 genes commonly involved in hematological malignancies was targeted using PCR enrichment with primers specific to these genes. The PCR products were sequenced using an Illumina NovaSeq sequencer (San Diego, CA, USA). The sequencing results were analyzed using SeqNext software (JSI, Ettenheim, Germany) and laboratory-developed pipelines (Sano Medical Laboratories, China).

### 2.4 RT-PCR

cDNA synthesis from total RNA was performed using random priming and the SuperScript™ IV reverse transcriptase (ThermoFisher). PCR amplification was then carried out using specific primers for *ETV6::SNCB* (forward primer *ETV6*: 5’-TCTTAAATGACCGCGTCTGGC and reverse primer *SNCB:* 3’-GACAGAATTGTGCTGCTGGTG, nested PCR forward primer *ETV6*: 5’-TTGGGGAGAGGAAAGGAAAGTG and reverse primer *SNCB:* 3’-CATACTCCTGATATTCCTCCTGGG) and *ETV6::SNCB::GPRIN1* (forward primer *ETV6*: 5’-TTCTTAAATGACCGCGTCTGGC and reverse primer *GPRIN1*: 3’-ATCCTGTGGGCAGAAGAAGG, nested PCR forward primer *ETV6*: 5’-TGGGGAGAGGAAAGGAAAGTG and reverse primer *GPRIN1:* 3’-GGGCTGGAATCCTTTTGAAGC). The PCR protocol consisted of one cycle at 94°C for 2 minutes followed by 30 cycles at 94°C for 30 seconds, 68°C for 30 seconds, and 72°C for 30 seconds, one cycle at 72°C for 1 minute, and final holding at 4°C. The 1st PCR product (0.5 µl) was used as template DNA for the nested PCR. The PCR products were then subjected to Sanger sequencing.

## 3 Results

### 3.1 *ETV6::SNCB* rearrangement in a B-ALL

**Figure 1:**
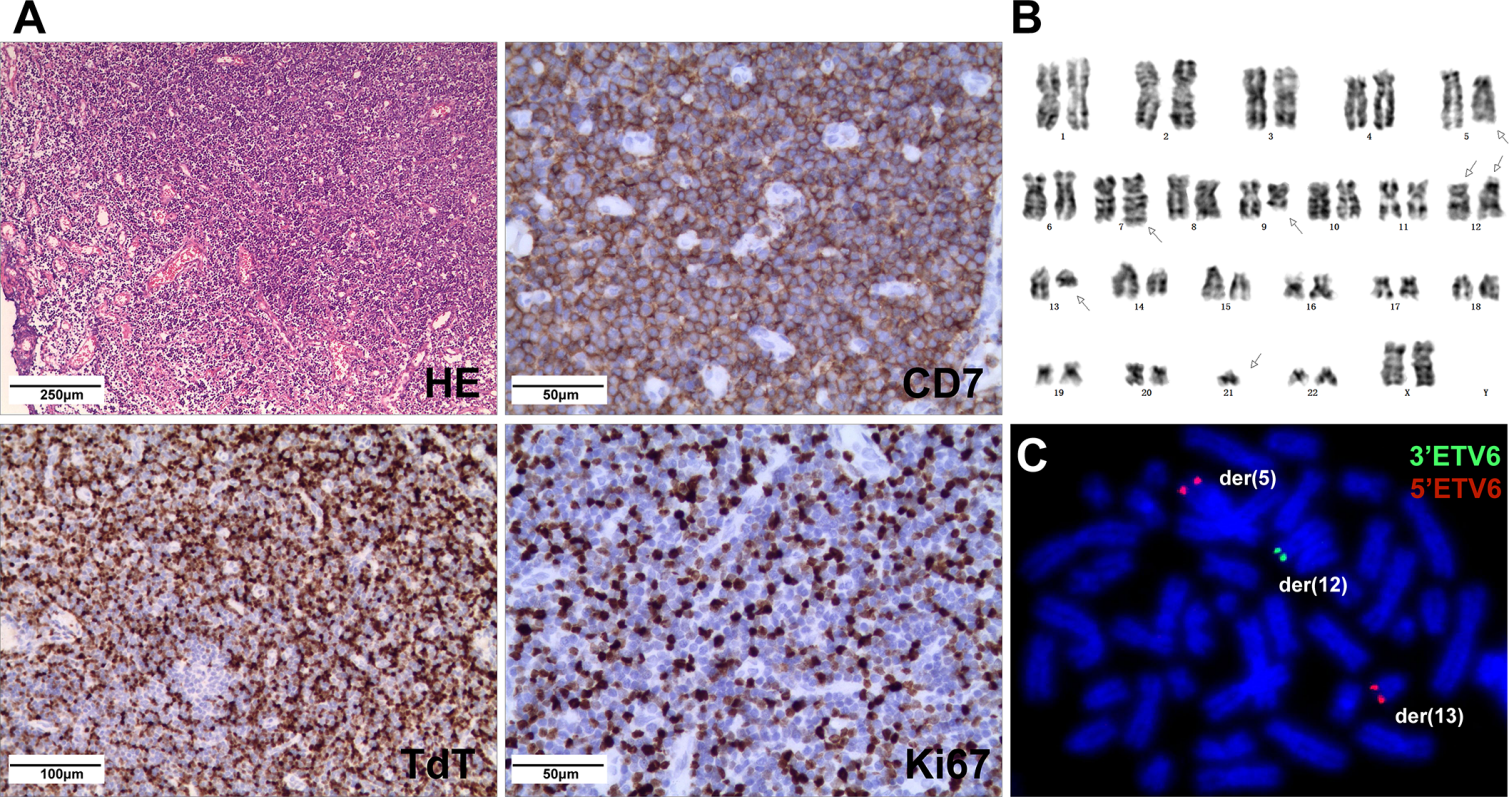
**(A)** Neck lymph node biopsy showed complete effacement of architecture with small lymphoblasts (HE stain) and were positive for CD7, TdT, and Ki67 (40%) by IHC. **(B)** The karyotype of the cultured bone marrow showed complex chromosome changes with both copies of chromosome 12p13 rearranged. **(C)** FISH analysis on abnormal metaphases with the *ETV6* break-apart probe revealed an *ETV6* rearrangement by the t(5;12), while the other *ETV6* allele had a deletion of the 3’*ETV6*, with the 5’*ETV6* translocated to der(13).

**Figure 2:**
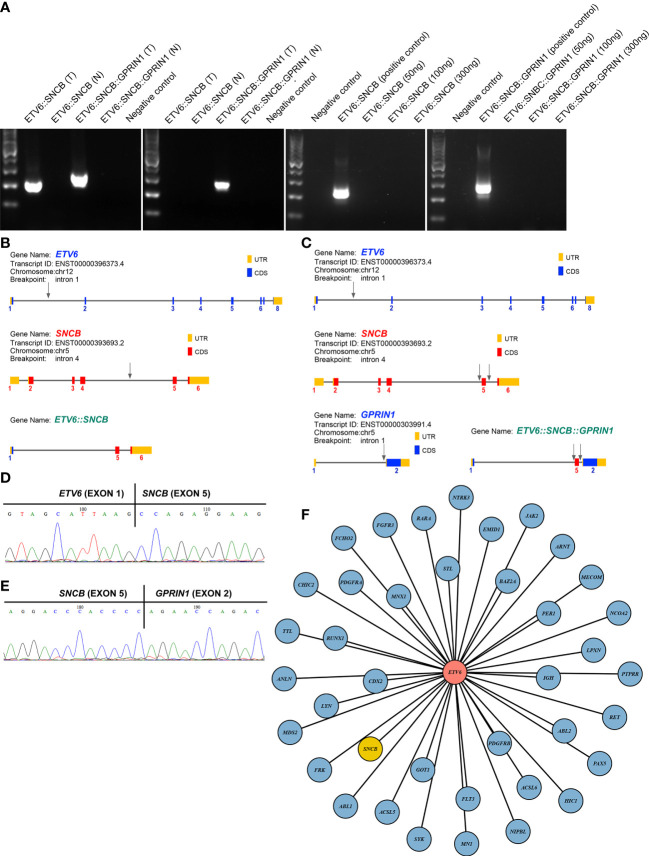
**(A)** RT-PCR amplified 2 fusion transcripts, *ETV6::SNCB* and *ETV6::SNCB::GPRIN1*, from the diagnostic bone marrow specimen, consistent with the RNA NGS finding (left panel). At day 19, only *ETV6::SNCB::GPRIN1* was observed, consistent with MRD (next to the left panel). At day 49, both fusion transcripts were absent from the patient’s bone marrow (right 2 panels). T: tumor bone marrow; N: normal bone marrow; Negative control: reactions without cDNA template; Positive control: tumor materials from the initial diagnostic specimen; **(B)** Exons and breakpoints (arrows) of the *ETV6::SNCB;* **(C)** Exons and breakpoints (arrows) of the *ETV6::SNCB::GPRIN1;* **(D)** Sanger sequencing of the RT-PCR for *ETV6::SNCB*; **(E)** Sanger sequencing of the *ETV6::SNCB::GPRIN1*; **(F)** Total 39 fusion partners of the *ETV6* in cancer.

A 5-year-old girl presented with a neck mass for 2 months. Physical examination revealed lymphadenopathy on both sides of her neck. Complete blood counts showed WBC 1.39 x 10^9^/L, Hb 91 g/L, PLT 111 x 10^9^/L, 31.7% neutrophils, 65% lymphocytes, 0% monocytes, 0% eosinophils, and 0% basophils. A neck lymph node biopsy showed an effaced node with diffuse infiltration of blasts positive for CD3 (dim), CD7, TdT, CD99, PAX5, Ki-67 (40%) and negative for MPO, CD20, CD21 (**Figure 1A**). The bone marrow smear revealed that 65% of the cells were blasts of various sizes with small amounts of blue-grey cytoplasm, dispersed nuclear chromatin, and inconspicuous nucleoli. Cytochemistry was positive for periodic acid-Schiff (PAS) and negative for myeloperoxidase. The bone marrow flow cytometry showed 58% of CD45+ cells with cCD3+, CD5+, CD7+, CD33dim, CD34dim, CD99+, CD1a-, CD2-, sCD3-, CD4-, CD8-, CD13-, CD19-, cCD79a-, MPO-, TdT-. Based on these results, a diagnosis of T-ALL was made. The karyotype analysis of her bone marrow showed a complex karyotype with 45,XX, t(5;12)(q35;p13), t(7;9)(q34;q22), add(12)(p13), der(13) del(13)(q14q32)t(12;13) (p13;q34), -21[16]/46, XX[4] (**Figure 1B**). Notably, both copies of chromosome 12p13 (*ETV6* locus) were rearranged. The FISH assays revealed that one locus of the *ETV6* was rearranged and the other was partially deleted at the 3’ end of *ETV6*. The rearranged 5’ *ETV6* was found at the 5q terminal, which was consistent with the translocation between chromosomes 5 and 12 (**Figure 1C**). To identify the *ETV6* fusion partner gene, a targeted RNA-NGS assay was conducted. This showed the presence of two in-frame fusion transcripts: the first was made up of the first exon of *ETV6* and the last two exons (exon 5 and 6) of *SNCB*, while the second contained sequences from three genes, including the first exon of *ETV6*, exon 5 of *SNCB*, and the last exon (exon 2) of *GPRIN1*. As the *GPRIN1* is located immediately 3’ to *SNCB*, the three-gene fusion transcript is believed to be a read-through product of the *ETV6*::*SNCB* rearrangement. The presence of both fusion transcripts was confirmed by RT-PCR with gene-specific primers and subsequent Sanger sequencing (**Figures 2A–E**). We believe this is the first fusion transcript involving 3 genes observed in the tumor.

The patient was enrolled in a clinical trial conducted by the Chinese Children’s Cancer Group (CCCG) and was treated with the CCCG-2020 protocol. This protocol involved a four-day pre-treatment phase with dexamethasone, followed by induction therapy combining dexamethasone, vincristine, daunorubicin, peg-asparaginase, cyclophosphamide, cytarabine, and 6-mercaptopurine. The patient also underwent additional early intensification therapy consisting of cyclophosphamide, cytarabine, 6-mercaptopurine, vincristine, and peg-asparaginase, followed by consolidation therapy with high-dose methotrexate, triple intrathecal therapy every other week, and daily 6-mercaptopurine for four courses. After one year, the patient remains in remission.

The therapeutic response was monitored using both *ETV6::SNCB* and *ETV6::SNCB::GPRIN1*. On day 19 of the treatment, the *ETV6::SNCB* disappeared but the *ETV6::SNCB::GPRIN1* remained positive in the bone marrow, which is consistent with minimal residual disease (MRD). However, on day 46 of the treatment, both *ETV6::SNCB* and *ETV6::SNCB::GPRIN1* had disappeared (as shown in **Figure 2A**). These results, obtained through RT-PCR, were consistent with the patient’s clinical remission, negative histology findings, and negative flow cytometry.

### 3.2 *LDLRAD3::SNCB* rearrangement in a lung squamous cell carcinoma

**Figure 3:**
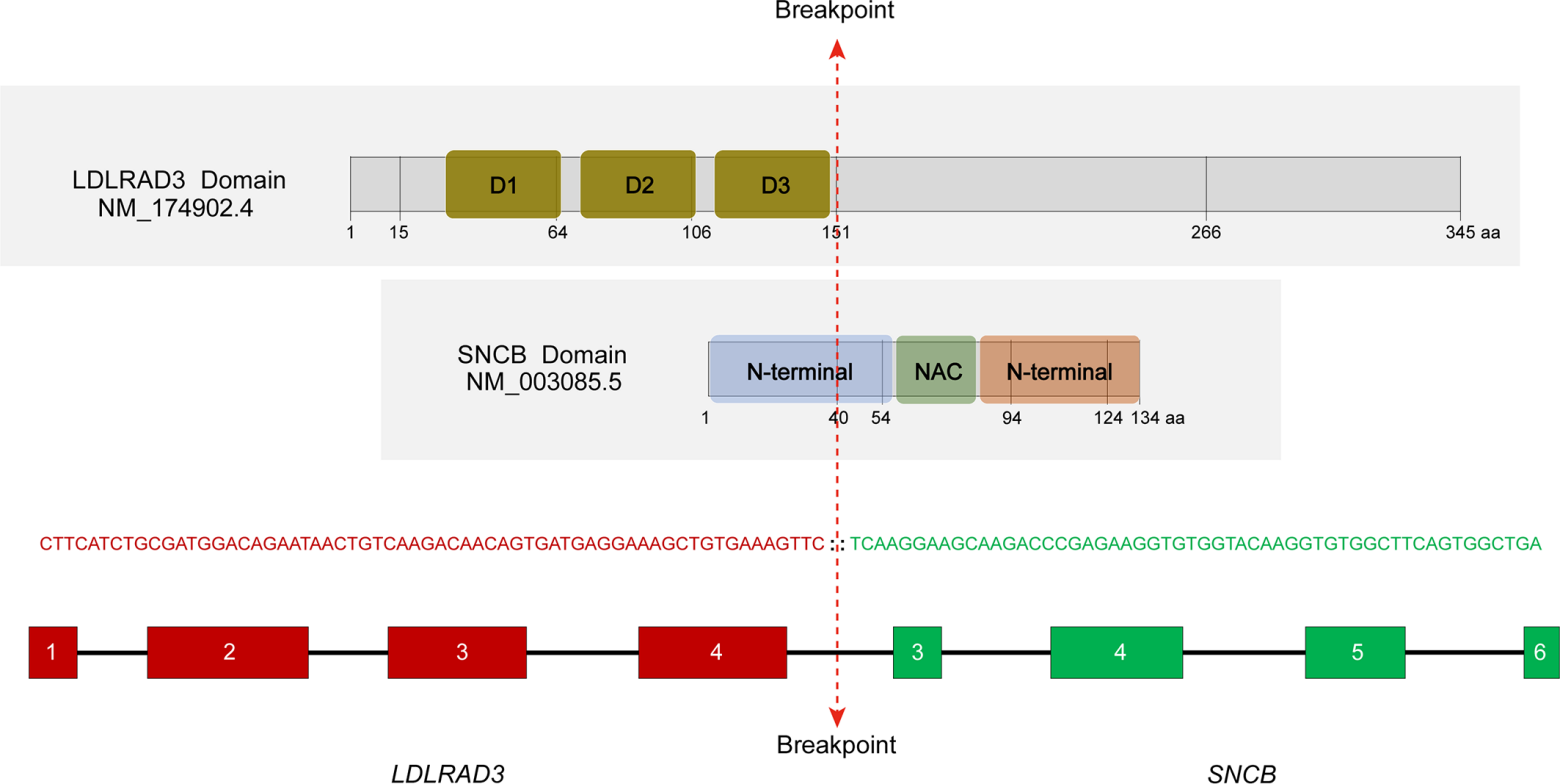
Characterization of the *LDLRAD3*::*SNCB* rearrangement. The location of breakpoints of corresponding proteins was marked. D1-3, domains 1-3; NAC, non-amyloid β component domain. RNA sequencing showed an in-frame fusion between *LDLRAD3* exon 4 and *SNCB* exon 3.

In a search of 10,565 cancer genomes from the TCGA database, a lung squamous cell carcinoma with the presence of *LDLRAD3::SNCB* fusion (TCGA-85-A50M-01A) was identified, among a total of 423 cases of lung squamous cell carcinoma. The patient was a 46-year-old male with a 15-year smoking history. The rearrangement involves the first four exons (exons 1-4) of *LDLRAD3*, which encodes low-density lipoprotein receptor class A domain containing 3, and the last four exons of *SNCB* (exons 3-7). The reading frame is intact, which encodes the domains 1-3 (D1-3) of the LDLRAD3 and the partial N-terminal, the NAC domain, and the C-terminal of the β-synuclein (**Figure 3**). The role of *LDLRAD3* in cancer has not been extensively studied, however, *LDLRAD3* is overexpressed in several types of cancers including breast cancer, glioma, prostate adenocarcinoma, skin cutaneous melanoma, testicular germ cell tumor, and pancreatic cancer (TCGA, data not shown). Similarly, circular RNA circ-*LDLRAD3* is upregulated in pancreatic cancer, lung adenocarcinoma, gastric cancer and is significantly associated with venous invasion, lymphatic invasion, and metastasis in pancreatic cancer (10).

## 4 Discussion

*ETV6* is a promiscuity gene that plays a role in the development of various tumors, such as leukemia, lymphoma, secretory carcinoma of the breast, infantile fibrosarcoma, congenital mesoblastic nephroma, and thyroid cancer. Our literature review uncovered 38 fusion partners of *ETV6*, with the *SNCB* being the 39th fusion partner as shown in **Figure 2F**. *ETV6* rearrangements can lead to oncogenesis through various mechanisms (11). One of the most common rearrangements, *ETV6::RUNX1*, is found in around 25% of pediatric B-ALL and leads to the dysregulation of genome-wide gene expression. The wild-type ETV6 protein has the ability to form dimers with ETV6::RUNX1, which reduces the transforming activity of ETV6::RUNX1 and mitigates the malignant process. However, in many tumors, the wild-type ETV6 is lost, which exacerbates the malignant process. In our case with the *ETV6::SNCB* rearrangement, we observed a similar loss of wild-type *ETV6*, which likely promotes cancer progression through a similar mechanism.

14-3-3s are a group of small proteins that play a crucial role in programmed cell death. α-synuclein binds to 14-3-3s, and both are present in Lewy bodies in patients with Parkinson’s disease. The interaction between α-synuclein and 14-3-3s can have both positive and negative effects on the fate of neurons. 14-3-3s serve as chaperones to reduce α-synuclein aggregation, providing neuroprotective activity, but α-synuclein can also sequester 14-3-3s and release the apoptotic BAD and BAX, leading to apoptosis. The potential oncogenic mechanism of the ETV6::SNCB or LDLRAD3::SNCB may involve the overexpression of the C-terminal synuclein driven by its fusion with either *ETV6* or *LDLRAD3*, both of which are highly expressed genes. This overexpression may bind and sequester 14-3-3s, compromising their anti-apoptotic signaling. Other mechanisms, including p53-dependent and Akt-related apoptotic pathways, may also be involved (12, 13).

The involvement of synucleins in cell proliferation is primarily seen in γ-synuclein. Overexpression of γ-synuclein leads to increased cell proliferation in H175 (squamous cell carcinoma) by binding and activating the AKT. γ-synuclein is a nicotine-responsive protein and is essential for nicotine-induced cancer cell proliferation (14). Diabetes is also a risk factor for lung cancer, and glucose-induced lung cancer cell proliferation is mediated by γ-synuclein (15). In ovarian cancer cell lines A2780 and OVCAR5, forced expression of γ-synuclein increased cell proliferation by activating the MAPK signaling pathway and attenuated the chemotherapeutic drug-induced apoptosis by blocking the c-Jun N-terminal kinase (JNK) signaling (8). The ETV6::SNCB only retains the C-terminal acidic region of β-synuclein, while the LDLRAD3::SNCB contains the partial N-terminal α-helix domain, NAC domain, and C-terminal acidic region of β-synuclein. Further investigation is needed to determine if these fusion proteins have different effects on cell proliferation and/or apoptosis.

In conclusion, we have reported the first cases of synuclein rearrangement in tumors. Further study of these chimeric proteins may provide insights into not only tumorigenesis but also the physiological function of β-synuclein, which is crucial to understand synucleinopathies.

## Data availability statement

The original contributions presented in the study are included in the article/supplementary material. Further inquiries can be directed to the corresponding authors.

## Ethics statement

Ethical review and approval was not required for the study on human participants in accordance with the local legislation and institutional requirements. Written informed consent to participate in this study was provided by the participants’ legal guardian/next of kin. Written informed consent was obtained from the individual(s), and minor(s)’ legal guardian/next of kin, for the publication of any potentially identifiable images or data included in this article.

## Author contributions

Project design: PX, SH and SX. Clinical studies: PX, SH, XB, JM and JZ. Laboratory work: NC, TS, XL, YW, XC and LJ. Result interpretation: PX, SH, XL, NC and SX. Draft manuscript: NC and SX. All authors contributed to the article and approved the submitted version.
